# Forensic Psychiatric Patients' Experiences of Personal Recovery: A Wilsonian Concept Analysis

**DOI:** 10.1097/JFN.0000000000000477

**Published:** 2024-02-03

**Authors:** Katja Lumén, Olavi Louheranta, Lauri Kuosmanen

**Affiliations:** **Author Affiliations:** 1 Department of Forensic Psychiatry, University of Eastern Finland, Niuvanniemi Hospital; 2 Department of Nursing Science, University of Eastern Finland.

**Keywords:** Concept analysis, forensic patients' perceptions, forensic psychiatry, recovery

## Abstract

Psychiatric patients' recovery processes have been studied rather extensively, and a relatively clear consensus on what recovery is already exists. We examined whether the personal recovery of forensic psychiatric patients varies from this definition. We conducted a concept analysis to assess the personal recovery of patients in forensic psychiatric hospitals based on 21 articles and then evaluated how our results compared with existing definitions on psychiatric and forensic recovery. On the basis of this comparison, we concluded that the personal recovery of forensic psychiatric patients does differ from that of other psychiatric patients. The recovery process of forensic psychiatric patients can be described through five themes: personal development and autonomy, social inclusion and normality, redemption and overcoming, future orientation and hope, and advancing process. The burden of a psychiatric disorder causes similar challenges, yet a criminal history and long hospitalization periods are distinctive issues for forensic patients, both of which can lead to severe alienation from society and deterioration of skills needed in life outside an institution. The results suggest that processing the criminal offense should be part of the care; furthermore, institutions should support forensic psychiatric patients in their reintegration into society and personal development. Identifying special recovery challenges can be useful when designing effective care and promoting the recovery of forensic patients. Thus, our results suggest that certain features of forensic psychiatric patients' recovery should be considered when planning their psychiatric care.

The literature describes various forms of care and activities that can promote the recovery processes of forensic psychiatric patients, but the core experiences of recovery are less often summarized. Nevertheless, there are certain studies that comprehensively describe both the patients' experiences of recovery in forensic hospitals and helpful forms of care (e.g., Clarke et al., 2016; Senneseth et al., 2022; Shepherd et al., 2016). The patient populations of forensic psychiatric hospitals differ from one country to another (Askola et al., 2022). When referring to forensic patients in this article, we mean patients who have offended. In the case of forensic patients, a criminal history and long-term psychiatric illness affect the patients' possibilities to pursue their own goals (Clarke et al., 2016; Drennan & Alred, 2012; Slade et al., 2014). The legal stipulations because of the forensic patients' offending history may affect their possibilities to leave the hospital (Askola et al., 2022). We conducted a concept analysis to define forensic patients' personal experiences of recovery and propose how personal recovery can be promoted in forensic psychiatric hospital care.

## Background

In psychiatry in general, the recovery paradigm emphasizes patient-centered care and the uniqueness of each patient's personal recovery toward a meaningful life and well-being (Lloyd et al., 2008; Schoppmann et al., 2021). Recovery is defined as a personal process that encompasses changes to help the person enjoy a satisfying life and receive help from others (e.g., Anthony, 1993; Schoppmann et al., 2021). Recovery can be perceived in several different ways, for example, as clinical based, service based, and personal or client based (Simpson & Penney, 2011). However, it is common for forensic hospitals to strike a balance between patient empowerment and institutional control during the care process (Schoppmann et al., 2021; Senneseth et al., 2022; Tomlin et al., 2018). Forensic hospitals typically treat forensic patients, but in some countries, psychiatric patients who are dangerous and so severely mentally ill that they cannot be treated in general psychiatry may be treated in a forensic hospital (Askola et al., 2022). In forensic hospitals, patients are detained in closed environments because of their mental illness and because they have offended or are at risk of offending (Livingston et al., 2013; Nijdam-Jones et al., 2015). Nurses need to balance between care and risk, and forensic patients need to deal with criminal responsibility, legal consequences, and self-determination (McKeown et al., 2016). The archetype of a forensic hospital traditionally serves as a place for “imposed” recovery, as it deprives patients of their freedom to choose their own path of recovery (McKenna et al., 2014). Lengthy treatment periods are typical for different kinds of patients in forensic hospitals (Askola et al., 2022).

In general, clinical, functional, social, and personal recovery can each be distinguished as a unique phenomenon. The concept of clinical recovery refers mostly to the mitigation of symptoms (Roosenschoon et al., 2019) and adequate utilization of psychiatric care (Lloyd et al., 2008; Slade, 2010). Functional recovery is related to improving performance in everyday activities, as assessed by both the patient and others (Lloyd et al., 2008). Social recovery is connected to social inclusion (Lloyd et al., 2008), which is especially challenging for forensic patients because of their criminal history, typical symptoms of long-term mental illness, long periods of hospitalization, and stigma associated with mental health disorders (Møllerhøj, 2021). Personal recovery focuses on the patient's experiences of recovery as a journey toward a meaningful life and was therefore the concept chosen for this study (Drennan & Alred, 2012; Slade, 2010).

The “CHIME” theoretical framework for recovery from mental illness was formulated over a decade ago. The acronym encompasses Connectedness, Hope and optimism about the future, Identity, Meaning in life, and Empowerment (Leamy et al., 2011). However, Van Weeghel et al. (2019) stated that the best results from CHIME can be obtained when it is applied to psychiatric care that meets certain contextual features; this need was the motivation for our study. We were interested in how the personal recovery of patients in forensic hospitals can be perceived when the care periods are long, the environment is highly restricted, and the patients are expected to be dangerous and are severely mentally ill (Askola et al., 2022). In the latter part of the article, we suggest how nursing practice should consider the typical features of this patient population and care environment.

Recently, the CHIME framework was expanded to the CHIME-S framework by Senneseth et al. (2022) to better fit the forensic context and highlight the importance of experiencing safety during forensic care. Shepherd et al. (2016) also studied forensic patients' recovery and provided an interpretation of the phenomenon. These researchers emphasized a personal sense of safety and linked this feature of care to experiences of a safe environment, facilities, and social interactions. They articulated this finding not as the core feature of recovery experienced by the patient but rather something that enables the recovery experience. Hope and social networks were presented as the second theme to express the importance of these factors during the recovery process. The third theme was work on identity, which describes how a patient's personal history (including both mental health disorder and criminal offenses) needs to be integrated into the recovering patient's life story for personal psychological development to take place.

Clarke et al. (2016) reported that forensic patients' perceptions of recovery and therapeutic measures are promoted through six themes: connectedness, sense of self, coming to terms with the past, freedom, hope, and health and intervention. The present article focuses on the concept of personal recovery experienced by forensic patients and aims to provide insight into the patient perspective. We considered the power imbalance between patients and staff in forensic psychiatry when formulating the methodology used and chose patients as the first source of information. Our methodological choice (concept analysis) indicates how we were interested in giving patients a voice, which is in line with the paradigm of recovery orientation. We also aimed at promoting nursing knowledge concerning care of forensic patients.

## Methods and Data Collection

### Concept Analysis

A concept is the cognitive representation of a phenomenon that can be detected as it is (Morse, 1995, 2017) or by observing feelings, experiences, or other events connected to it (Fitzpatrick & McCarthy, 2016). Concept analysis can be used to examine a concept in the case that a consensus or clarification of the use and meaning of the chosen concept is needed (Fitzpatrick & McCarthy, 2016). This type of methodology is also beneficial for outlining how a certain concept is used in a new context or different field of science (Fitzpatrick & McCarthy, 2016; Tofthagen & Fagerstrøm, 2010; Wilson, 1963). The use of a concept can modify the perceptions of the concept and understanding the phenomenon that it describes. In this sense, the concept and phenomenon that it describes are intricately intertwined (Morse, 2017; Wilson, 1963). The use of concepts varies in time (Walker & Avant 2014, p. 184), and it affects our understanding and attitudes as well as behavior (Tofthagen & Fagerstrøm, 2010) toward the phenomenon that the concept describes. Understanding what patients in forensic settings perceive as personal recovery promotes staff's capability of relating to the patient experience and mutual understanding during the care process. Evaluating the results of concept analysis assists theory development, understanding the elements of nursing, and the improvement of professional practice (Fitzpatrick & McCarthy, 2016; Morse, 2017; Walker & Avant, 2014). Concept analysis confronts the challenge of abstract definitions by distinguishing essential characteristics (Walker & Avant, 2014) and constructing model cases (Morse, 1995; Wilson, 1963).

The Wilson approach includes more phases than most other concept analysis methods, and some of the other phases differ from each other (e.g., Risjord, 2009; Tofthagen & Fagerstrøm, 2010). Wilson's (1963) work was intended to aid students to think more thoroughly about the features and traits of the phenomena that different concepts describe. The fact that Wilson did not intend to create a concept analysis method has been a source of criticism toward his work. Other methods have since been developed “to correct limitations” of Wilson's analysis process (Hupcey et al., 1996, p. 185). However, these methods have lost some of the nuances that made Wilson's analysis more profound (Hupcey et al., 1996). The Wilson method links the concept to the context in a stronger way than, for example, Walker and Avant's (2014) method as it considers the situational factors and the environment (in Phase 8, Social Context, and Phase 9, Underlying Anxiety) and includes the practical results and results in language (Phases 10 and 11). In our study, the restrictive environment in forensic hospitals and forensic patients' history of mental illness as well as offending affect the recovery experiences of patients and cause underlying anxiety. Hupcey et al. (1996) point out that Wilson's method is dialogical, as the flow between different phases of analysis deepens the scope of results. In our analysis, we implemented this idea when we interpreted the meaning of different features from the perspective of the forensic patients. As such, the Wilsonian method of concept analysis ensured a deeper understanding of the phenomenon of personal recovery among forensic psychiatric patients. The different phases of the analysis performed in this study are typical of Wilson's method (Wilson, 1963) and are presented in more detail in Table 1. We later refer to these phases in the text as we describe the analysis process and report the results of our study.

**TABLE 1:** Phases of Wilson's Concept Analysis Applied in This Study

Phase	Outcome
1. Isolating questions of concept	Personal recovery of a forensic psychiatric patient
2. Right answers	A patient's personal experiences instead of helpful forms of care or views of others
3. Model cases	Including the essential features
4. Contrary cases	Contradicting features
5. Related cases	Some essential features excluded
6. Borderline cases	Some resemblance to the pure case
7. Invented cases	All examples in this article
8. Social context	Forensic care in a closed environment
9. Underlying anxiety	The double burden and challenges of a criminal history and psychiatric disorder
10. Practical results	Understanding the experiences of forensic patients
11. Results in language	Comprehensible, useful expressions of the concept

### Data Collection

**FIGURE 1:**
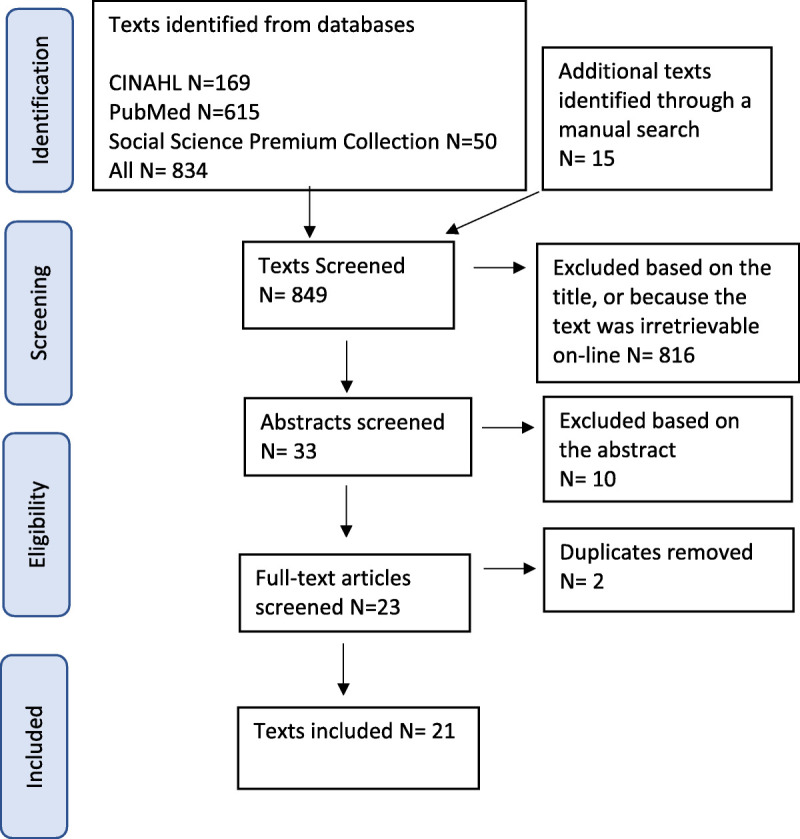
Search strategy.

The research question for our concept analysis can be defined as “What do the forensic psychiatric patients' experiences of recovery entail?” (Phase 1). We initially chose a preliminary definition of the concept (forensic psychiatric patients' experiences of personal recovery) and then collected data from peer-reviewed literature (Phase 1). The concept analysis aided us in defining the concept, and this definition could be compared with previous more general definitions of psychiatric recovery and definitions of forensic recovery. Searches were conducted in the CINAHL (EBSCO), PubMed (MEDLINE), and Social Science Premium Collection (ProQuest) databases. Search terms were as follows: in CINAHL, recovery OR rehabilitation OR healing AND forensic psychiatry OR forensic care OR high security; in PubMed, recovery AND forensic hospital; and in Social Science Premium Collection, forensic psychiatr* AND ti(recover*). Our search criteria for data in these databases were published in the English language, published in a peer-reviewed journal, original article, and the research having included a description of recovery from the perspective of a patient with severe mental illness in a forensic or high-security setting. We also included one book as a source of data because the contents focused on the chosen patient population and recovery, and we performed a manual search of the reference lists of identified relevant studies (see Figure 1). We ensured all the references included in the data portrayed forensic hospital settings, patients with a history of offending or who had been labeled dangerous, and descriptions of recovery from the patient perspective.

### Data Analysis

To increase the quality of the research, it was important to find a genuinely pure research question (concept) and avoid questions of value. During data collection, we needed to refocus our research question and simplify the concept we were analyzing (Phase 2). More specifically, we rejected definitions portraying what enables recovery or what recovery means from the perspective of staff or others. It is not expedient to list all possible features of the concept in this report. In our analysis, the essential features were found and grouped into themes and cases. We tolerated “hum noises” in the data, remembering the uniqueness of personal experiences, and some features were dismissed from the results because of their very low incidence. For example, in a book chapter in our data, the patient answered a question concerning their experience of recovery: “Right medication.” This unique feature can be understood rather as a helpful form of care than an essential feature of the personal recovery experience.

We extracted the possible features of personal recovery of forensic patients from the original texts and gathered them into a list based on previous definitions by Anthony (1993) and Leamy et al. (2011). These definitions are more general descriptions of psychiatric recovery and were used to test whether our findings would fit the previous descriptions or differ from it. We constructed a phrase: “Recovery is 1) *personal and unique*, 2) a *progressive journey with many stages and obstacles* towards 3) *a good life* which is 4) *more than absence of symptoms* or *overcoming the burden of disease* and includes 5) *citizenship and integration into society* through 6) *development of emotions and perceptions*.” This proved to be an inaccurate way of organizing our findings, and we proceeded to create our own mode of categorization that would illuminate the forensic patients' experiences more accurately. We investigated the prevalence of different features identified in the chosen literature to define the typical nuances of forensic recovery experiences. We read through the list of features several times and, during the analysis process, found special features and unique meanings concerning forensic patients. Features resembling each other were grouped together until five themes entailed the typical nuances of personal recovery of patients in forensic care.

## Results

The collected data (see Table 2) were categorized into five themes that describe the personal recovery experiences of forensic patients and emphasize the uniqueness of this group of psychiatric patients. Although we found similarities with previous descriptions of recovery in general (Anthony, 1993; Leamy et al., 2011) and secure or forensic recovery (Clarke et al., 2016; Senneseth et al., 2022; Shepherd et al., 2016), we accentuate the different meanings of recovery for patients in forensic care. Our five overarching themes were as follows: personal development and autonomy, social inclusion and normality, redemption and overcoming, future orientation and hope, and advancing process.

**TABLE 2:** Incidence of Five Themes Among the Identified Studies

	Personal development and autonomy	Social inclusion and normality	Redemption and overcoming	Future orientation and hope	Advancing process
Aga et al. (2019)	x	x	x	x	x
Barnao et al. (2015)	x		x		
Barsky & West (2007)	x	x			
Chandley et al. (2014)	x	x	x	x	x
Chandley & Rouski (2014)	x		x	x	
Ferrito et al. (2012)	x	x	x	x	x
Frayn et al. (2016)	x			x	
Glorney et al. (2019)	x	x	x		
Livingston et al. (2013)	x	x			
McKenna et al. (2014)	x	x		x	
McKeown et al. (2016)	x	x	x	x	x
Mezey et al. (2010)	x	x	x	x	x
Miles et al. (2012)	x	x	x		x
Moore et al. (2012)	x	x	x	x	x
Nijdam-Jones et al. (2015)	x	x	x		
Olsson et al. (2014)	x		x		
Pollak et al. (2018)	x	x	x	x	
Pouncey & Lukens (2010)	x	x	x		
Simpson & Penney (2011)	x	x			
Stanton & Simpson (2006)	x	x	x		x
Vandevelde et al. (2017)	x	x		x	

**FIGURE 2:**
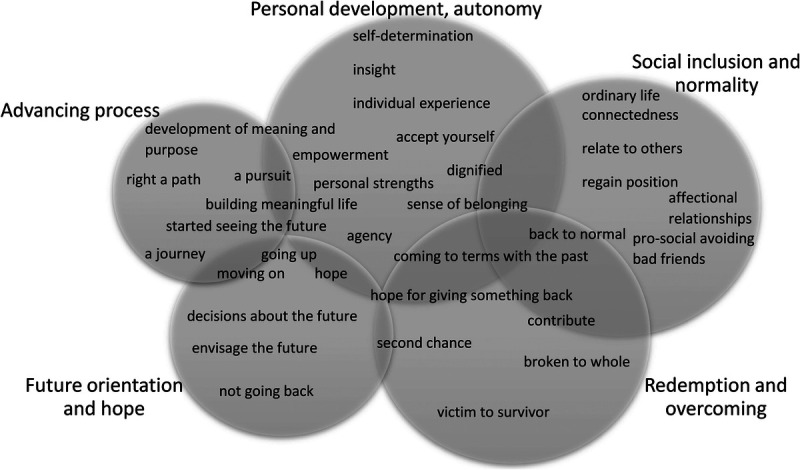
Features and themes describing forensic psychiatric patients' personal recovery, with the size of a circle reflecting the amount of features belonging to a certain theme.

The collected data showed clear associations with these themes. Several original articles emphasized themes differently. The features in the data provided coherent examples of how patients experience their personal recovery process. It is noteworthy that some of our themes overlap, and certain features can be organized under two or more themes; this should not have a significant impact on the research because all of these features are a part of the concept under study. This overlapping serves as evidence that the personal recovery of the forensic patient is a complex phenomenon. The reasoning underlying the grouping of features into the five themes is depicted in Figure 2 and in the following case examples.

### Personal Development and Autonomy

All of the identified articles mentioned how a changing self-image and the development of new skills are a part of recovery. Examples of this included active coping (Moore et al., 2012), utilizing strengths (McKenna et al., 2014), experiencing different emotions (Chandley et al., 2014), mastery over challenges (Simpson & Penney, 2011), feeling in control (Nijdam-Jones et al., 2015), empowerment (Pollak et al., 2018; Pouncey & Lukens, 2010; Simpson & Penney, 2011), interests and dreams as aspirations (Aga et al., 2019), making sense of past experiences as coming to terms with the past (Aga et al., 2019), acceptance and working through denial (Moore et al., 2012), autonomy (Aga et al., 2019; Barnao et al., 2015; Livingston et al., 2013; Nijdam-Jones et al., 2015) and independence (Barsky & West, 2007), agency (Nijdam-Jones et al., 2015; Vandevelde et al., 2017), self-management and self-efficacy (Frayn et al., 2016), self-confidence and self-reflection (Olsson et al., 2014), a sense of achievement (Aga et al., 2019; Simpson & Penney, 2011), feeling better (Mezey et al., 2010; Miles et al., 2012), feeling good and important (Olsson et al., 2014), and being worthy (Mezey et al., 2010). Especially in the case of processing past life events, the offending history is a special feature for forensic patients. Empowerment and building autonomy and agency in a strictly closed environment are also special challenges for patients in forensic hospitals.

### Social Inclusion and Normality

This theme primarily described integration into society after hospitalization, with the articles citing relevant activities such as social inclusion (Miles et al., 2012), social bonding (Nijdam-Jones et al., 2015), networking (Aga et al., 2019), life as a citizen (Aga et al., 2019; Livingston et al., 2013; McKenna et al., 2014), being accepted (Aga et al., 2019), making a useful contribution (Aga et al., 2019; Chandley et al., 2014; Pollak et al., 2018, Simpson & Penney, 2011), companionship as in close affectional relationships (Nijdam-Jones et al., 2015), connecting with others (Aga et al., 2019; Pollak et al., 2018), being able to relate to people (Aga et al., 2019; Moore et al., 2012; Nijdam-Jones et al., 2015; Pollak et al., 2018; Stanton & Simpson, 2006; Vandevelde et al., 2017), joining the work environment or enrolling in education (Aga et al., 2019, Simpson & Penney, 2011), finding a home (Glorney et al., 2019), becoming a useful and contributing member of society (Chandley et al., 2014), internalizing social norms (Nijdam-Jones et al., 2015), and becoming average (Moore et al., 2012). This theme was discussed in 17 articles. Finding their own place in the society as full members can be especially challenging for forensic patients because of their long care in an institution. Normalcy can be perceived through this wish, to have the same possibilities as others who live without a history of mental illness and the role of a patient in a forensic or high-security hospital. Double stigma of mental illness and offending are present in forensic patients' lives.

### Redemption and Overcoming

We found descriptions of overcoming the burden of disease and history of offending in 15 references. Redemption and overcoming were discussed in terms of criminal going straight and badness to redemption (Chandley et al., 2014), finding a new identity separate from the past (Glorney et al., 2019), helping instead of hurting (Aga et al., 2019), hope for giving something back (Ferrito et al., 2012), working on processing the crime (Pollak et al., 2018), understanding that an offense never leaves you (Moore et al., 2012), and having an identity beyond being a patient or offender (Glorney et al., 2019). Although this theme was not the strongest, it clearly depicts special challenges for forensic recovery, the need for extra work on identity, and processing traumatic life events.

### Future Orientation and Hope

In our sample, 11 references clarified how this theme is important to personal recovery. More specifically, the articles described how patients can gain future orientation and hope by pursuing a satisfying life (Chandley et al., 2014), feeling as though life is worth living (McKenna et al., 2014), not going back to previous ways (Moore et al., 2012), letting go of the past (Aga et al., 2019), looking into the future (Chandley & Rouski, 2014), envisaging the future (Ferrito et al., 2012), and perceiving hope as something to live for (Moore et al., 2012). Orientation toward the future instead of clinging to the past experiences is a common theme for all psychiatric patients, but for forensic patients, not going back also means no more offending, no recidivism.

### Advancing Process

The classic definition of recovery among psychiatric patients highlights that this is a continuous process. Our findings show that the recovery of forensic patients differs from the classic definition to some extent as only eight of the articles explicitly stated that recovery is a continuous, ongoing process. These articles commented that personal recovery involves building a life (Nijdam-Jones et al., 2015), is a process (Chandley et al., 2014), can be considered as a journey (McKeown et al., 2016), is lifelong (Chandley et al., 2014) rather than an outcome one arrives at (Pouncey & Lukens, 2010), is nonlinear (Aga et al., 2019), and encompasses one's own path (Vandevelde et al., 2017) or clearly moving on (McKeown et al., 2016). Perhaps the processual nature of recovery seems less important for patients in closed forensic institutions because of the typically long treatment periods. It has been reported that the length of stay at the forensic hospital can be unclear to the patients (e.g., Nijdam-Jones et al., 2015). During the long hospitalization, it can be difficult to start planning life outside the hospital and to see the inpatient period as a phase of the recovery process. This theme is closely linked to the previous one.

Wilson's (1963) method includes constructing cases to clarify the use of a concept. The cases (Wilson's Phases 3–6) created in this study are reported in the next paragraph. We only used invented cases (Phase 7). Social contextualization (Phase 8) and the question of underlying anxiety (Phase 9) were strongly present in our choice of concepts and phenomenon (Wilson, 1963). These contextual nuances were an important part of the analysis process. Social contextualization and the underlying anxiety are related to forensic patients' recovery context because the forensic hospital represents a high-security, closed institutional environment that involves limited possibilities for patients to choose social connections (e.g., Reavey et al., 2019; Tomlin et al., 2018). The recovery process of forensic patients includes understanding their history as “offender patients” (Nijdam-Jones et al., 2015) and managing a psychiatric condition (e.g., Reavey et al., 2019), which can cause double stigmatization. Moreover, the care provided by staff involves a certain tension in balancing care and risk assessment (Clarke et al., 2016; Wilson, 1963). We considered the social contextualization and underlying anxiety as important factors in the forensic context and regarded these during different phases of the analysis process and interpretation of the findings. This way of conducting the analysis is typical for Wilson's method.

In this study, the cases were formulated as discussed below.

#### The Model Case

“I am moving forward toward my own goals, and I find it a promising journey. I am accompanied by my loved ones, and I am finding new roles and possibilities in society as perhaps almost anyone could. I acknowledge that previous crimes will influence my future, as does my psychiatric condition, but I have gained self-awareness, responsibility, and confidence. I do feel worthy, less impaired, and more capable.” This model case encompasses the essential features of personal recovery for forensic patients. The patient describes hope, self-determination, social networking, autonomy, overcoming adversity, finding new meaning in life, and trust in the future. The patient has found new capability of controlling their own life, conduct, interactions, and future. This statement acknowledges that the recovery process is a journey; moreover, instead of feeling hindered by their psychiatric illness and criminal history, the patient is finding new possibilities to look forward to.

#### The Contrary Case

“I feel limitations and restrictions and am somehow disconnected from the rest of the world. The symptoms have finally alleviated to some extent and the doctor should be happy. I do not know what I could hope for or be entitled to, either now or in the future. Perhaps I can return to my old life soon and forget about this whole mess caused by others.” In the contrary case, the patient does not feel in control of their life and has trouble being hopeful for new or better things. Furthermore, they have shifted the responsibility for their situation (a mess caused by others) to someone else, and the patient seems to lack motivation and purpose in recovering. As such, the essential features are not present in the contrary case (Wilson, 1963).

#### The Related Case

“I understand that what happened [the criminal offense] was because of my condition. I hope never to be and feel that way again. I am somewhat hopeless and lonely in this situation and do not have the necessary skills to proceed. Fortunately, I am getting support and assistance from my friends and the hospital staff. Maybe I can understand things better and find a new path in life.” In this case, the patient is interested in getting better but still doubtful about whether it will happen or how long it will take. This patient is connected to people, which creates hope. However, they are still lacking empowerment and strong motivation, but they are beginning to overcome the burden of their personal history. This example shows some of the elements of personal recovery but is not a pure case. It is a description of the personal development of a patient who is at the start of the recovery process (Wilson, 1963).

#### The Borderline Case

“I am getting some new ideas for the future. I am no longer as anxious as I was before, but sometimes I feel bitter and worry about how my life is going to turn out. This keeps me from experiencing true hope, and I do not quite feel worthy of happiness and a place in the outside world.” This patient is anticipating that something good and worthy will happen in the future but is still feeling insecure. The personal history is not yet integrated into this patient's life story and sense of self; as such, there has been no redemption or adopted responsibility. Although the symptoms have resolved, it remains unclear whether the psychiatric challenges have been accepted and understood. In this borderline case, the patient describes the features of recovery on a rather superficial level and experiences difficulties in self-expression (Wilson, 1963).

The last phases of Wilson's concept analysis (Phases 10 and 11) include the practical results and clarifying the concept in the studied context, that is, “results in language.” Our results can help practitioners to better understand forensic psychiatric patients' experiences, which would benefit interventions aimed at designing appropriate care for the forensic environment. It can help the staff address important issues during the therapeutic process. Nevertheless, it is important to note that the analysis is not generalizable to all cases, as recovery has been described as a subjective and unique experience that changes over time (Clarke et al., 2016; Slade et al., 2014; Wilson, 1963). In the best-case scenario, forensic psychiatric care can help a patient recover from their illness and mitigate the risk for recidivism by education about self-management approaches (Senneseth et al., 2022).

## Discussion

Both the CHIME framework (Leamy et al., 2011) and the previous definitions of forensic recovery (Clarke et al., 2016; Senneseth et al., 2022; Shepherd et al., 2016) are applicable to the forensic context, yet our findings promote a deeper understanding of the concept of personal recovery from the patient's perspective. The theme personal development and autonomy resembles previous definitions of forensic recovery, that is, identity, meaning in life, and empowerment, as well as identity work and developing a sense of self (Clarke et al., 2016; Senneseth et al., 2022; Shepherd et al., 2016). Moreover, the definition used in this study categorizes meaning in life and empowerment within personal development and autonomy. As such, the definition links our findings to the recovery paradigm, which emphasizes how a meaningful life and well-being are connected to personal development (Lloyd et al., 2008; Schoppmann et al., 2021). Our theme of social inclusion and normality is closely related to connectedness (Clarke et al., 2016; Leamy et al., 2011; Senneseth et al., 2022) and social networks (Shepherd et al., 2016), and encompasses special features in the case of forensic patients. Our definition contains two distinct themes—” redemption and overcoming,” which was also considered important in the work by Clarke et al. (2016), and “advancing process.”

The CHIME terms connectedness and hope and optimism about the future reflect, to some extent, the features of future orientation and hope and social inclusion and normality in our definition. Normalcy plays a significant role in the recovery of all psychiatric patients and is particularly important to forensic patients. Hope and looking forward to the future are commonly noted as central characteristics of recovery (e.g., Clarke et al., 2016; Leamy et al., 2011; Shepherd et al., 2016). In this way, previous knowledge of psychiatric recovery emphasizes this theme more than our findings from a population of forensic psychiatric patients. It was surprising that only 11 references of the identified 21 touched upon the theme of future orientation and hope, which is a central feature of the general recovery paradigm. Instead, several studies of forensic psychiatric patients included descriptions of long treatment periods and time seemingly “standing still.” This *presenteeism* can be a challenge, especially in forensic care (Reavey et al., 2019). Perhaps the processual nature of the forensic recovery process was rarely mentioned in the identified studies because of presenteeism. The duration of hospital care can seem infinite from the patient perspective (Nijdam-Jones et al., 2015). This infiniteness is associated to the arbitrary length of stay conditional upon the patient's mental state and risk assessment, both of which are evaluated by the staff.

## Conclusions

Our concept analysis included five overarching themes that contain the dimensions of recovery for forensic psychiatric patients. In comparison with previous definitions of personal recovery, our results stress that the recovery process of forensic psychiatric patients involves some special features. Each of the different dimensions of recovery (clinical, functional, social, and personal) was present in our findings and interacts in the patients' experiences in the clinical environment. The dimensions also overlap and change in time.

### Implications for Clinical Forensic Nursing Practice

The results of this study provide several insights into the development of forensic nursing practice (Phase 10). First, supporting a patient's capacity to integrate their personal history into their life story and creating a sense of self should be critical elements in forensic nursing. Therapeutic work that focuses on the experience of the criminal offense, as well as its consequences for the patient and others, should be the primary form of support for forensic patients with a psychiatric illness. Second, inclusion and belonging can be promoted by providing possibilities for reorientation into society both during and after hospitalization, which is typically long-term. The boundaries of the hospital should be considered a risk for alienation, and protective activities should be organized through work with the patients' families and possibilities for engaging in professional activities and/or education. Moreover, patients should be afforded the opportunity to train for the skills required for common everyday activities outside the institution. Third, personal development and finding meaning in life should be a key part of the therapeutic relationship between nurses and forensic patients. It is important to state that the goal of treatment should not be a symptom-free life, but rather a life without the excess limitations caused by a psychiatric condition and criminal history. As such, supporting a patient's capacity to create new self-awareness, self-management skills, and self-efficacy will improve their sense of agency and add meaning to their life. This is critical to helping a patient get involved in social activities, which are a large part of reducing recidivism.
